# Barriers of Care to Ovarian Cancer: A Scoping Review

**DOI:** 10.7759/cureus.40309

**Published:** 2023-06-12

**Authors:** Zehra Rizvi, Kiran C Sharma, Viktor Kunder, Adrian Abreu

**Affiliations:** 1 Osteopathic Medicine, Nova Southeastern University Dr. Kiran C. Patel College of Osteopathic Medicine, Fort Lauderdale, USA; 2 Obstetrics and Gynecology, Broward Health Medical Center, Fort Lauderdale, USA

**Keywords:** gynecology and obstetrics, ovarian cancer screening, risk-reducing bilateral salpingo-oophorectomy, barriers to care, ovarian cancer

## Abstract

Ovarian cancer is a leading cause of female cancer-related deaths, but patients continue to be diagnosed late. This subjects them to disease progression and possible death due to lack of early detection, despite earlier stage detection improving survival rates by significant percentages. Early detection and access to care are closely related. However, many barriers to high-quality care exist for patients and the majority of patients do not receive recommended care according to ovarian cancer treatment guidelines. In order to improve care for ovarian cancer patients and decrease healthcare disparities in accessing equitable care, it is important to acknowledge the current gaps in patient knowledge, healthcare availability, and physician practice. This scoping review explores the available evidence on ovarian cancer to identify these barriers to care in the effective treatment of ovarian cancer. Using both inclusion and exclusion criteria, results from the initial literature search were screened by the authors. After quality assessment and screening for relevance, 10 articles were included in the final review. The following themes emerged as barriers to care: hospital and physician-patient volumes, geographic distance from care facilities, patient and physician education, and demographic factors. Many patients were found to not receive guideline adherent care due to various barriers to care, whereas guideline adherent care was independently associated with factors such as patient proximity to a high-volume hospital, White race, and higher socioeconomic status. Several areas were identified as potential for increased patient and physician education, including treatment complications and patient resources. The evidence found that certain socioeconomic groups and racial minorities are often at higher risk for guideline non-adherent care. Additionally, the evidence showed a further need for physicians and healthcare providers to be provided with resources for post-cancer treatment, follow-up, and patient education. The findings of this review will provide health experts and patients with better insight into what barriers may exist so that guideline-adherent care can be better advocated for and met.

## Introduction and background

Ovarian cancer remains a serious issue in the United States, with ovarian cancer being a leading cause of female cancer-related deaths, thus making it the most lethal female genital tract cancer [1-5]. A vast majority of patients are still diagnosed at advanced stages, resulting in a worse prognosis for patients [1-2]. Ovarian cancer localized to the ovaries (stage 1) has a 90% cure rate, but less than 20% of cases are stage 1 at the time of diagnosis [1,5-6]. This means that patients are being diagnosed late and further subjected to disease progression and possible death due to a lack of early detection [2]. Previous analysis and research suggest that early-stage detection of ovarian cancer could improve survival rates by as much as 30% [3,6].

Early detection and access to care are closely related. Recommendations are to have a high index of suspicion in any female patient from 40 to 70 years of age with persistent, unexplained gastrointestinal complaints [2]. However, many barriers exist to high-quality care, and most patients do not receive recommended care according to ovarian cancer treatment guidelines [7]. Racial minority and socioeconomic statuses are linked to worse outcomes in many aspects of healthcare and have similar effects on ovarian cancer outcomes [7-9]. Minority patients are less likely to be aware of genetic testing for ovarian cancer detection than their White counterparts, leading to more advanced stages of diagnosis [8]. The availability of physicians and hospitals that take on larger volumes of ovarian cancer cases is also significant in treatment outcomes and is again impacted by race and sociodemographic status [7,9]. 

However, even independent of race and socioeconomic status, patients are still being diagnosed at advanced stages with worse survival rates. Lack of guideline adherence, availability and knowledge of specific genetic testing, geographical distance, and access to risk-reducing procedures are additional barriers that lead to disparities in, and access to care for patients. In order to improve care for ovarian cancer patients and decrease healthcare disparities in accessing equitable care, it is important to acknowledge the current gaps in patient knowledge, healthcare availability, and physician practice. This scoping review explores the available evidence on ovarian cancer to identify these barriers to care in the effective treatment of ovarian cancer.

## Review

Methods

Following PRISMA guidelines, a comprehensive electronic search was conducted to identify articles discussing socioeconomic, racial, and demographic factors as barriers to ovarian cancer. The studies used in this scoping review were found through searches in PubMed, Medline, CINAHL Complete, Cochrane Database of Systematic Reviews, and Biomedical Reference Collection: Comprehensive. According to the criteria described below, studies published from 2002 to 2022 were included. Based on the inclusion and exclusion criteria, three independent and blind reviewers conducted the selection process.

Search Strategy

Using the databases mentioned earlier, a computer-assisted literature search was conducted to identify studies that meet the following inclusion criteria: (1) articles published between 2002 and 2022, (2) full texts in English, and (3) articles discussing socioeconomic, racial, and demographic factors as barriers to ovarian cancer. Excluded articles described gynecologic cancers other than ovarian cancer, did not focus on barriers to care or were published before 2002. The search was conducted in December 2022 and yielded 58 results.

Identification of Studies

The following search terms were used for all databases: (gynecology) AND (ovarian cancer) AND (barriers to care)

Data Extraction

All researchers assembled pertinent information on a data log that included the author and year, study type, sample size, and major findings after screening and applying the inclusion criteria to the articles they got from the databases. A Google Docs spreadsheet contained the results. The information was then organized, and each article was thoroughly discussed to see if it met the criteria for inclusion and the standards for quality. Discussions were used to settle disagreements.

**Figure 1:**
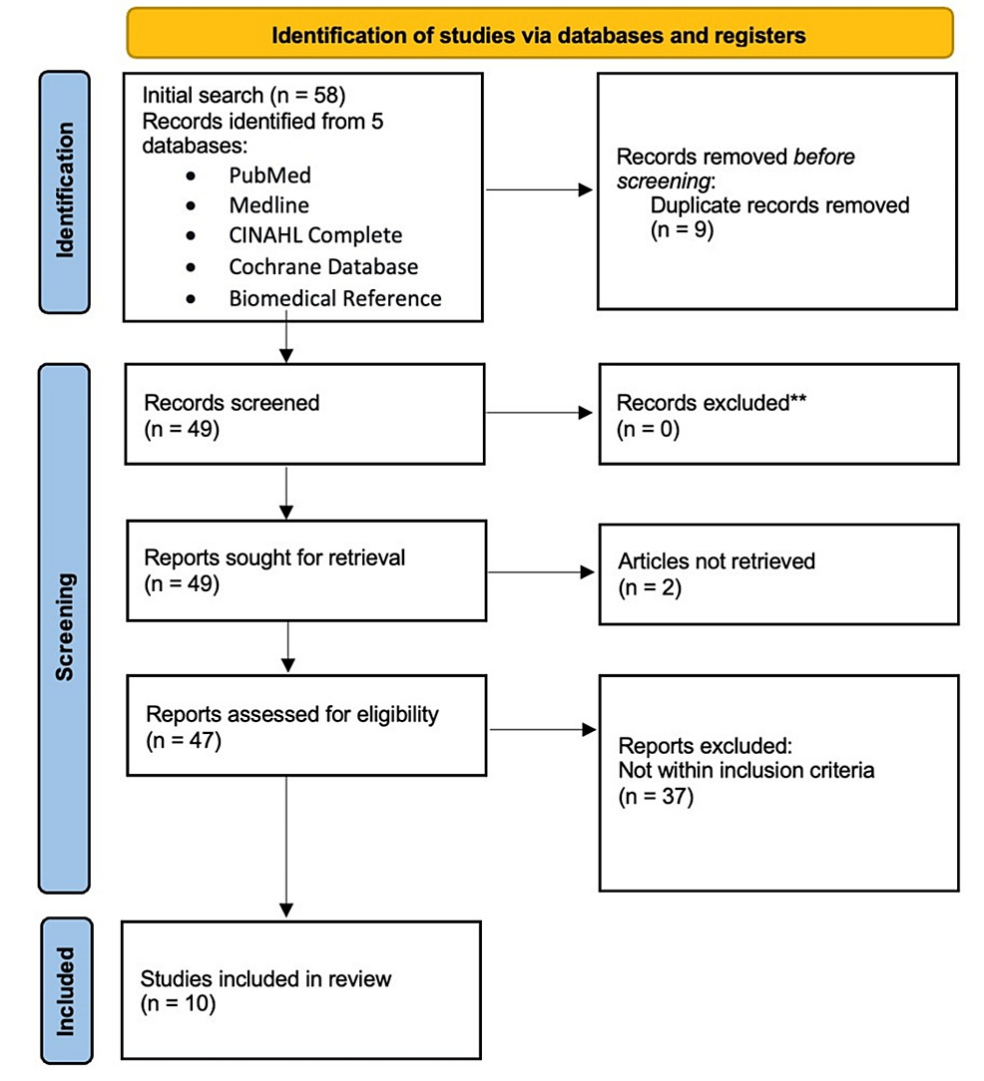
PRISMA flow diagram depicting the study selection process PRISMA: Preferred Reporting Items for Systematic Reviews and Meta-Analyses

Based on the provided search criteria, the initial search yielded 58 articles. Nine duplicates and an additional 31 articles were filtered out because they contained variables that did not meet the inclusion criteria or were not accessible. The remaining papers underwent a quality assessment procedure following the screening process. Ten articles were chosen for this review. These articles used data from national registrations and surveys (Figure 1).

Results

A total of 10 studies were identified using the research selection pathway described in Figure 1. Table 1 reports the characteristics of the studies included in this scoping review, which included studies that utilized methods such as evidence-based reviews, surveys, and database extractions.

**Table 1:** A summary of important findings and general characteristics of the included reviewed studies LVH = low volume hospital, LVP = low volume physician, SES = socioeconomic status, HVH = high volume hospital, HVP = high volume physician, BRCA = BReast CAncer gene, RRBSO = risk-reducing bilateral salpingo-oophorectomy, OS = opportunistic salpingectomy

Author	Study design	Purpose	Measures	Key findings
Bristow et al., 2013 [7]	Database extraction	To characterize the impact on ovarian cancer survival for high-volume hospitals (HVH) and physicians.	Factors that determine whether the patient will be treated in a high- or low-volume treatment center.	Low-volume hospitals (LVH) and low-volume physicians (LVP) were associated with the lowest ovarian cancer-specific survival. Black patients were more likely to be treated at an LVH, while Hispanic patients were the least. Higher socioeconomic status (SES) was associated with HVH) and high-volume physicians (HVP). White patients and those with private insurance had the most access to HVP, while Black patients and non-private insurance had the least. Medicare was significantly associated with LVH.
Lacour et al., 2008 [8]	Survey	To investigate the factors that play a role in women who have ovarian cancer.	Patients' knowledge and willingness to undergo genetic testing	Less than half of the patients diagnosed with ovarian cancer were aware of BRCA (BReast CAncer gene) genetic testing, with the Black population being the least aware. Lower education was also directly correlated to less knowledge about BRCA genetic testing. Almost every patient was willing to receive genetic testing, whether it benefited themselves or their family. Most patients were willing to receive testing at a 20% copayment, but only 25.1% were willing to pay the total cost.
Bristow et al., 2014 [9]	Database extraction	To determine how geographical location impacts advanced-stage ovarian cancer care adherence to the National Comprehensive Cancer Network (NCCN) guidelines in relation to race and socioeconomic status.	Proximity to HVH, race, geographical location, distance traveled to receive care, and socioeconomic status for patients with stage IIIC/IV epithelial ovarian cancer.	Geographical location of ≥ 50 mi from an HVH was independently associated with an increased risk of non-adherent care, whereas traveling ≥ 20 mi to receive care was associated with adherent care. Higher SES was associated with being in closer proximity to HVH. The White race was most likely to be within a 20 mi radius of an HVH, with the Black race most likely to have the least access.
Temkin et al., 2018 [10]	Evidence-based review	To outline health disparities, especially for the Black race in receiving care for ovarian cancer.	Disparities specific to gynecologic malignancies including ovarian cancer.	Fewer than 50% of women diagnosed with ovarian cancer receive guideline adherent care, which correlates with survival. Factors for non-guideline-adherent care include low SES, the Black race, and a low-volume surgeon and hospital. Additional at-risk factors include non-White race, Medicaid insurance, lack of insurance, and non-English fluency.
Miller et al., 2018 [11]	Evidence-based review	To outline healthcare discrepancies in women with ovarian cancer.	Factors that contribute towards improved mortality and quality of life in women with ovarian cancer.	There is no known effective screening method for ovarian cancer in patients without a known increased risk. Compared to White women, Black women have been found to have higher mortality from ovarian cancer, and their median survival time is also about one year shorter. Minority and low-income ovarian cancer patients do not obtain hospice care at the same rate as White and higher-income patients.
Temkin, 2022 [12]	Journal review	To assess the current practices and barriers to quality care for ovarian cancer.	Provides comprehensive recommendations using a multidisciplinary approach about how to best manage ovarian cancer.	Compared to White patients, Black and Hispanic patients are less likely to obtain care that complies with guidelines, with or without a gynecologic oncologist consult. They are also under-represented in ovarian cancer clinical trials. In addition, patients with public insurance or who are uninsured are less likely to receive guideline-adherent care than those with private insurance or managed care. These historically under-represented racial and ethnic groups and those with low socioeconomic status face an overall survival disadvantage due to these inequities in care delivery.
Bristow et al., 2020 [13]	Retrospective database extraction	To determine how disparities in access to high-performing hospitals impact the care in ovarian cancer.	Mortality associated with hospitals with different levels of observed/expected ratios and how disparities contribute to where the patient is managed.	Hospitals with higher observed/expected ratios were significantly associated with longer survival hood than hospitals with low or intermediate observed/expected ratios. Factors that included worse survival outcomes included being of the Black race, not being married, having either Medicaid or no insurance, and being of lower SES. Hispanic patients with Medicare had a negative predictive value for access to a high observed/expected ratio hospital.
Hickey et al., 2020 [14]	Survey and interview	To determine gaps in information for patients who receive RRBSO.	Qualitative and quantitative responses about the inadequate information received relating to RRBSO.	Nearly all patients were well informed about how risk-reducing bilateral salpingo-oophorectomy (RRBSO) can reduce the risk of ovarian cancer and the severe nature of an ovarian cancer diagnosis. The main barrier to RRBSO was the loss of fertility, followed by symptoms associated with surgical menopause (hot flushes, loss of libido, osteoporosis, sleep disturbances, memory and mental health problems, loss of muscle tone, and increased risk of heart disease). More than half of the pre-RRBSO participants reported inadequate information about the management of sexual health, cancer checkups, bone health, and how to manage menopausal symptoms. In the post-RRBSO group, one woman commented that ‘‘the effects of surgery were much harder than I was led to believe’’ and that they needed more help with ‘‘managing the long-term effects of having little estrogen.’’ There was a significant difference in the post-surgical advice concerning hormone therapy, making the patient feel like they got conflicting information from different specialists.
Hickey et al., 2020 [15]	Cross-sectional survey	To determine barriers that may lead high-risk women not receiving RRBSO for ovarian cancer.	Responses from high-risk patients and healthcare providers about RRBSO.	Reasons for women not wanting to undergo RRBSO include surgical menopause and physical changes. Almost half of the high-risk participants did not receive written resources about RRBSO, but the information varied significantly with the women who did. Almost all the healthcare participants made referrals for high-risk women considering RRBSO, but there was no explicit coordination or pathway to direct these referrals. In addition, healthcare professionals lacked resources for optimizing long-term health, hormone therapy, postsurgical menopause, and access to multidisciplinary care.
Gelderblom et al., 2021 [16]	Mix-method study	To emphasize improving counseling on benefits and risks of OS.	Qualitative and quantitative responses about the concerns of receiving or performing OS.	The most common barrier for patients who received hysterectomy or sterilization to refuse opportunistic salpingectomy (OS) was the “low lifetime risk of ovarian cancer in the general population and the residual risk after OS.” Additional barriers included not knowing the disadvantages, no insight about the size of the surgery (referring to potential complications), and a lack of knowledge about OS before being consulted. Most healthcare professionals briefed a concern about insufficient skills in performing OS via the vaginal approach and not having additional counseling material. Furthermore, professionals are concerned that there needs to be more evidence regarding risk reduction and long-term effects such as surgical menopause.

Equity of Care

Less than half of women diagnosed with ovarian cancer receive the recommended care due to various factors [9,11], which include low SES, low volume surgeons and hospitals, race, type of insurance and lack of insurance, and non-English fluency [10,12]. Low-volume hospitals (LVH) and low-volume physicians (LVP), defined here as hospitals and physicians who see a small number of ovarian cancer patients, had lower ovarian cancer survival. Additionally, Black patients were more likely to receive care at one of these facilities. Aside from race, Medicare and lower SES were also associated with a significant increase in LVP care, whereas factors that resulted in high-volume physician (HVP) care included being White and having private insurance [7,12]. 

The observed/expected ratios for adherence to ovarian cancer treatment guidelines at hospitals were another important factor influencing the level of care. Hospitals with high observed/expected ratios had disease-specific survival times of 68.6-79.3 months, compared to hospitals with low observed/expected ratios with survival times of 44.8-50.8 months. Non-Hispanic Black race, Hispanic race, unmarried status, Medicaid or no insurance, and low SES all predicted lower patient survival outcomes. Even after controlling for all variables, it was found that hospitals with high observed/expected ratios were associated with higher survival rates [13]. Moreover, it was found that patients in the highest socioeconomic status quintile were about 2.5 times more likely than those in the lowest socioeconomic status quintile to visit a hospital with a high observed/expected ratio [13].

The geographical location additionally played an important role in whether an individual would receive guideline adherent care. Guideline adherent care was independently associated with patient proximity to a high-volume hospital (HVH). Individuals with higher SES were associated with being in closer proximity to HVHs [9]. This same trend was seen in race, with patients of the White race having the highest access and those of the Black race having the lowest access [9].

Education

High-risk patients reported being well-informed about cancer risk reduction from risk-reducing bilateral salpingo-oophorectomy (RRBSO), post-operative infertility, and the seriousness of ovarian cancer diagnoses. However, many patients reported being misinformed about managing sexual problems, mental health, check-ups, bone and heart health, hormone therapy, and menopausal symptoms. Regarding hormone therapy, one respondent included, "I felt I got conflicting information from different specialists in relation to hormone therapy," and "I see a different doctor every time and hear different things about my treatment plan" [14].

Medical professionals were also poorly informed about how to care for women with ovarian cancer [15-16]. Nearly half of the high-risk women (women with close family members diagnosed with ovarian cancer) were not provided with written resources about RRBSO. In addition, despite needing additional referrals to other healthcare professionals, there was no clear pathway to standardize who should receive these referrals. Medical professionals also requested more information on RRBSO complications. Healthcare respondents lacked resources for optimizing long-term health, hormone therapy, postsurgical menopause, and access to multidisciplinary care [15]. 

Additionally, only a small percentage of patients were aware that BReast CAncer gene (BRCA) testing was available. Black patients were the least aware of genetic testing options, and a similar pattern was observed for patients with lower levels of education [8]. 

Barriers to Treatment Procedures

Although RRBSO has been known to reduce ovarian cancer fatalities by 95%, the main barrier for high-risk patients to receive an RRBSO was the loss of fertility, followed by symptoms associated with surgical menopause (hot flushes, loss of libido, osteoporosis, sleep disturbances, memory and mental health problems, loss of muscle tone, and increased risk of heart disease) [14-15].

In low-risk individuals, there was no practical method for screening for ovarian cancer [11]. When these low-risk patients were offered to have an opportunistic salpingectomy (OS) performed along with a hysterectomy or sterilization, the most important barrier for both patients and medical professionals to receive or perform an OS was the "low lifetime risk of ovarian cancer in the general population and the residual risk after OS." Additional barriers patients reported included a lack of knowledge about the disadvantages of OS, no insight about the potential complications and health impacts associated with the surgery, and never hearing about the possibility of OS prior to consultation [16].

Most hysterectomies and sterilizations were performed through the vaginal approach, and most healthcare professionals reported lacking the skills to perform OS via that method [16]. In addition, healthcare professionals stated that there was insufficient evidence of its effectiveness concerning risk reduction and a lack of knowledge about the long-term effects of OS, such as the onset of surgical menopause [16].

Discussion

This scoping review aimed to identify and discuss barriers to medical care concerning ovarian cancer. Following a thorough review and analysis of the ten included studies, we determined several barriers to care, such as low SES, LVH and LVP, race, level of patient education, geographic distance from care facilities, patient education on treatment options by physicians, physician education on treatment options, and lack of information on risk-reducing procedures.

One main barrier identified in multiple studies concerned the availability of appropriate ovarian cancer providers. Access to these individuals can be extremely limited and was even more pronounced for patients of racial minorities and low SES [7,9,12-13]. Institutions with more physicians could provide more care, which resulted in higher patient survival rates [7]. For income and racial disparities specifically, ovarian cancer patients of lower SES and non-White races were less likely to receive hospice care compared to their counterparts [11]. These inequities lead to an overall survival disadvantage for many patients with risk factors for lower quality of care [12].

Location and access to care are often tied to other risk factors such as SES and race. We found that geographic distance from HVHs was once again associated with low SES and racial minorities such as Black and Hispanic patients [13]. Additionally, geographical distance’s link to increased non-adherent care, in turn, further lowers survival rates in these patients and creates another survival disadvantage. Non-guideline adherent care is due to a mix of factors, such as medical comorbidities, lack of physicians, and risk factors for inequities, but the bottom line is that increasing adherence to guideline-accepted treatment methods is essential in increasing survival rates across the board [13].

As is the case in many aspects of healthcare, patient education is another extremely valuable tool. Lack of patient education by physicians about treatment options and steps in treatment can lead to worse outcomes. For example, the life-saving RRBSO procedure has been known to significantly reduce ovarian cancer fatalities [15], and while patients do report being educated about this option, many patients report not being educated on post-surgical outcomes, which affects patient decisions about electing for this surgery. Additionally, because patients do not feel adequately educated about complications, many decline the surgery due to concerns about infertility and surgical menopause [14-15]. Part of this lack of patient education can be attributed to healthcare providers being unaware of or being uncomfortable in their knowledge of post-surgical outcomes and care [15]. Another risk-reducing procedure called opportunistic salpingectomy (OS) presents with similar barriers, with a large percentage of patients reporting unawareness of post-surgical health impacts [16]. As such, it is clear that despite risk-reducing treatments being available, their effectiveness on the general patient population for ovarian cancer is significantly reduced due to improper patient and provider knowledge of the risks of these procedures.

Furthermore, many physicians rely on a high index of suspicion to proceed with ovarian cancer care. However, this may become a treatment barrier as some patients who may be seen as low-risk do not receive screening and thus do not get diagnosed until later stages, leading to worse survival outcomes. One example is seen with evaluating CA-125 values; CA-125 is increased in ovarian cancer patients, but the value is difficult to assess without a baseline CA-125 value from the patient first [6]. Another example is seen in BRCA mutations [8,14-15]. Those with the BRCA mutation have higher incidence rates of ovarian cancer, but some patients with high-risk BRCA mutations were unaware that BRCA testing even existed [8,14-15]. This lack of awareness was worse among minority patients [8], which further worsens mortality risks in this population. 

Limitations of included studies

Each study has a significant sociological aspect, and data was collected in different ways. Some studies used surveys, while others mixed qualitative and quantitative data, making comparison difficult and introducing recall bias. Additionally, studies examining guideline-adherent care made extrapolations based on their chosen set of guidelines. As there are several treatment guidelines, depending on location and scope of practice, results about guideline adherence, and the guidelines themselves may vary.

Limitations of the review process

During the review process of each article, some full-text articles could not be accessed and had to be dropped from the review process altogether. Articles predating 2002 were excluded from our scoping review, which may have limited our findings of additional barriers.

Implications for research and practice

The studies used in this review found significant barriers regarding ovarian cancer care. From racial minorities and poor socioeconomic status to basic patient education, many blockades exist and interrupt the road to care. This review found ten studies that met the inclusion criteria; further peer-reviewed research is needed on this topic. The primary purpose is to make patients and providers aware of the issues many women face when requiring life-saving interventions to reduce some of those risks. Further focus should also be on understanding how costs can be cut and how patient education can be better materialized and distributed so that all patients receive guideline-adherent, equitable care.

## Conclusions

This scoping review sought insight into barriers to ovarian cancer care. Extracted data revealed how social, economic, and educational factors may affect medical delivery. Findings proposed that members of a disadvantageous socioeconomic group may be left without the medical attention they deserve or need. Many reviewed studies also stated that adherent-based care is challenging to follow and worse in patients of lower socioeconomic status and racial minorities. This review also showed a clear need for physicians and healthcare providers to be better educated in post-cancer treatment, follow-up, and patient education. The findings of this review will provide health experts and patients with better insight into what barriers may exist and how they may be curtailed for more efficient ovarian cancer management and guideline-adherent care.
